# Oxidative Stress in Cardiovascular Diseases

**DOI:** 10.3390/antiox15060735

**Published:** 2026-06-10

**Authors:** Ernesto Martínez-Martínez, Roberto Palacios-Ramírez

**Affiliations:** 1 Departamento de Fisiología, Facultad de Medicina, Universidad Complutense de Madrid–Instituto de Investigación Sanitaria Gregorio Marañón (IiSGM), 28040 Madrid, Spain; 2 Ciber de Enfermedades Cardiovasculares (CIBERCV), Instituto de Salud Carlos III, 28220 Madrid, Spain; 3 Unidad de Excelencia Instituto de Biomedicina y Genética Molecular (IBGM), Universidad de Valladolid—CSIC, 47003 Valladolid, Spain

Cardiovascular diseases remain one of the leading causes of mortality worldwide, representing a major challenge for both healthcare systems and biomedical research [1]. Moreover, these disorders exert profound effects on the heart, promoting structural alterations that ultimately lead to functional impairment. A comprehensive understanding of the mechanisms involved in cardiovascular remodeling and injury across different pathological settings is essential for the identification of early disease biomarkers and the development of novel therapeutic strategies. In this context, oxidative stress is widely recognized as a key driver of inflammatory processes and fibrosis [2], thereby contributing significantly to the progression of cardiovascular damage. Given its high energetic demand, the heart relies predominantly on mitochondrial oxidative phosphorylation for ATP production, making cardiac tissue particularly vulnerable to disturbances in redox homeostasis [3]. Therefore, the study of redox pathways involved in cardiovascular damage and remodeling has attracted increasing scientific interest over recent decades, as redox imbalance can induce oxidative damage to lipids, proteins, and DNA, thereby contributing to cellular dysfunction and the progression of cardiovascular disease [4].

This Special Issue presents a curated collection of eight contributions—comprising comprehensive reviews and original research—that illuminate the current frontiers of redox biology in clinical practice. Martinez et al. (Contribution 1) provide a sophisticated analysis of mitochondrial ROS (mtROS) dysregulation in the context of heart failure with preserved ejection fraction (HFpEF). Unlike heart failure with reduced ejection fraction (HFrEF), HFpEF is often driven by systemic inflammation and microvascular dysfunction. The authors elucidate how mtROS serves as a critical signaling node that leads to Titin hypophosphorylation and increased myocardial stiffness. Furthermore, they discuss the “pleiotropic” antioxidant effects of standard pharmacological agents, such as SGLT2 inhibitors and ARBs, which may stabilize the mitochondrial membrane potential even when their primary clinical indication is metabolic or hemodynamic.

Complementing this, the review by Singh et al. (Contribution 2) explores the survival mechanisms under hypoxic conditions, focusing on the Hypoxia-Inducible Factor (HIF) signaling pathway. In the setting of Ischemia-Reperfusion (I/R) injury, the rapid influx of oxygen during reperfusion paradoxically triggers a massive ROS torrent. The authors detail how HIF-1α stabilization acts as a master regulator, attempting to shift metabolism from oxidative phosphorylation to glycolysis to minimize further ROS production. This “metabolic switch” is highlighted as a primary target for future cardioprotective therapies designed to limit infarct size.

The clinical management of coronary artery disease (CAD) remains a double-edged sword. While revascularization is essential, the reperfusion phase often exacerbates myocardial damage. Ghanta et al. (Contribution 3) synthesize the molecular insights into I/R injury, emphasizing the role of nicotinamide adenine dinucleotide phosphate (NADPH) oxidase and the xanthine oxidase system as secondary sources of superoxide. Their work underscores the failure of traditional “blanket” antioxidant trials and calls for more specific, compartment-targeted redox interventions.

A pivotal original research article by Castillo et al. (Contribution 4) bridges the gap between oxidative stress and innate immunity. By investigating patients undergoing cardiopulmonary bypass (CPB), the authors demonstrate that the surgical stress and extracorporeal circulation trigger the activation of the NLRP3 inflammasome. This activation is tightly coupled with oxidative markers, particularly in patients with pre-existing heart failure. Their findings suggest that the NLRP3-ROS axis could serve as a perioperative biomarker to identify patients at higher risk for post-surgical cardiac low-output syndrome, potentially justifying the use of anti-inflammatory or redox-modulating agents in the surgical setting.

Cardiovascular health cannot be decoupled from systemic metabolic status. Caturano et al. (Contribution 5) provide an in-depth review of the diabetic heart, where chronic hyperglycemia creates a “toxic” oxidative environment through the polyol pathway and the formation of Advanced Glycation End-products (AGEs). The authors meticulously detail how AGEs bind to their receptors (RAGE), activating NF-κB and promoting a pro-thrombotic state. A significant portion of their discussion is dedicated to lifestyle modifications, arguing that physical exercise and nutritional interventions are not merely supportive but are foundational “redox therapies” that can restore the Nrf2-mediated antioxidant response.

In a similar vein of systemic pathology, Quevedo-Abeledo et al. (Contribution 6) explore the heightened cardiovascular risk in Systemic Lupus Erythematosus (SLE). Their study identifies serum 3-nitrotyrosine—a footprint of peroxynitrite-mediated damage—as a robust marker for atherosclerosis in this population. By showing that 3-nitrotyrosine levels correlate with carotid plaque presence, the authors provide clinical evidence that protein nitration is a key driver of accelerated vascular aging in chronic inflammatory diseases.

The final theme of this issue focuses on practical interventions. Seth et al. (Contribution 7) contribute a rigorous systematic review and meta-analysis on the effects of Eicosapentaenoic Acid (EPA) and Docosahexaenoic Acid (DHA). By analyzing high-quality clinical trials, they conclude that Omega-3 fatty acids significantly ameliorate heart failure outcomes through the modulation of the lipidome and the subsequent reduction in systemic oxidative stress markers. Their work provides a much-needed evidence-based foundation for the use of these fatty acids in modern cardiac rehabilitation.

Finally, Mauriello et al. (Contribution 8) offer a critical perspective on the synergy between heart failure therapies and the prevention of atrial fibrillation (AF). They argue that the “electrical remodeling” characteristic of AF is fundamentally rooted in “redox remodeling.” By reviewing how drugs like SGLT2i and Sacubitril/Valsartan influence the atrial oxidative substrate, the authors advocate for an integrated pharmacological approach that treats heart failure and its arrhythmic consequences as a single, redox-driven entity.

The diverse contributions in this Special Issue collectively signal a paradigm shift. We are moving away from viewing oxidative stress as a simple imbalance of “good vs. bad” molecules and toward a nuanced understanding of “redox signaling.” The identification of specific biomarkers like 3-nitrotyrosine and the targeting of mitochondrial pathways represent the next step in precision cardiology. However, further experimental studies and well-designed clinical trials are required to achieve a deeper understanding of the contribution of oxidative stress to cardiovascular diseases and to facilitate the development of novel therapeutic strategies for these highly complex pathological conditions.
